# Is It Possible to Predict an Athlete’s Behavior? The Use of Polar Coordinates to Identify Key Patterns in Taekwondo

**DOI:** 10.3389/fpsyg.2019.01232

**Published:** 2019-05-29

**Authors:** Cristina Menescardi, Coral Falco, Isaac Estevan, Concepción Ros, Verónica Morales-Sánchez, Antonio Hernández-Mendo

**Affiliations:** ^1^AFIPS Research Group, Department of Teaching of Musical, Visual and Corporal Expression, University of Valencia, Valencia, Spain; ^2^Department of Sport, Food and Natural Sciences, Western Norway University of Applied Sciences, Bergen, Norway; ^3^GIEPAFS (Grupo de Investigación en Educación para una Actividad Física Saludable), Department of Motricity and Teaching in Physical Education, Universidad Católica de Valencia “San Vicente Mártir”, Valencia, Spain; ^4^Department of Social Psychology, Social Work, Social Anthropology and East Asian Studies, University of Málaga, Málaga, Spain

**Keywords:** taekwondo, behavioral patterns, Olympic Games, observational methodology, mixed methods

## Abstract

Match analysts and sport psychologists can help elite athletes develop planned competition strategies from a technical-tactical perspective by analyzing of previous performances. The aim of this study was to analyze the behavioral patterns used to score points in the 2012 London Olympic Games by a mixed observational methodology and polar coordinate analysis. This analysis is a representation of four quadrants of the relationships between focal behavior [i.e., scoring, (SC)] and conditioned behaviors as key factors in taekwondo before and after focal behavior of two lags (-2, +2). A total of 151 combats, with a total of 24,940 actions were analyzed by the Taekwondo Observational Tool, which consists of 24 categories grouped into seven criteria (tactics, techniques, kicking zone, laterality, kicking leg, guard, and score). Our analysis confirms 49 significant associations (21 in females and 28 in males) between different types of scoring actions (SC1: to the trunk, SC2: to the trunk with a previous spin, SC3: to the head and SC4: to the head with a previous spin) and a variety of technical-tactical aspects. Females SC1 after cut, direct attacks with circular techniques to the chest, with back right leg; SC2 after direct attacks to the head, and SC3 after cuts, posterior counterattacks with the back leg. Males SC1 after spin anticipate counterattack with back leg and dodges, SC2 after simultaneous counterattacks (SCAs) to the head, SC3 after cuts and posterior counterattacks with back leg while SC4 after blocks and SCAs in close guard with the forward right leg. The observed relationships provide objective data regarding successful behavioral patterns, and are important for coaches and psychologists to train and develop psychological strategies to prepare athletes. For instance, they can be used to individualize training sessions, including visualization of specific combat situations. Coaches and psychologists could use these findings for specific tasks related to technical-tactical improvement of scoring effectiveness or defensive strategies against these specific actions.

## Introduction

Psychology has a growing interest in the study of natural contexts and spontaneous behavior to discover and analyze behavioral patterns (Anguera et al., 2018). Analysis in natural contexts is especially important in elite sport for planning future competitive strategies. Previous performances can be visualized to detect underlying structures and behavioral patterns, assuming that similar patterns are usually repeated (Hernández-Mendo and Anguera, 2001, 2002). The analysis of natural contexts and sports behavioral patterns is also important for planning psychological training (i.e., visualization) in a wide range of competitive behaviors. There is evidence that visualization training activates the same muscles with almost the same intensity as real training (Folland and Williams, 2007). This analysis has been used in the mixed method approaches (for a review of a different studies see Anguera and Hernández-Mendo, 2014, 2015), which combine elements from both qualitative and quantitative research approaches (Johnson et al., 2007). Mixed method approaches are characterized by using systematic observation and qualitative data collection, stringent data quality controls and quantitative analysis, in a combination of qualitative and quantitative methods (Anguera et al., 2018).

Systematic observation has been used to collect information on key parameters in taekwondo based on their frequency of occurrence as tactics (Menescardi and Estevan, 2017), techniques (González, 2011; O’Donoghue, 2010), the kicking zone (Tornello et al., 2014), laterality or the kicking leg (Tornello et al., 2014), guard (López-López et al., 2015) or score (Tornello et al., 2014). The frequency (number of occurrences) provides the basic information on the behavioral tendencies. However, considering the order parameter takes the analysis one step further by providing not only the frequency, but also information on the order in which the behaviors occur (Anguera et al., 2018), so that strategic decisions regarding technical-tactical aspects can be made (Hernández-Mendo and Anguera, 2001). However, this information is difficult to transform into practical training applications. Relevant information has to describe tactics in a specific technique, toward a specific zone and with a given laterality in order to score a specific number of points.

**FIGURE 1:**
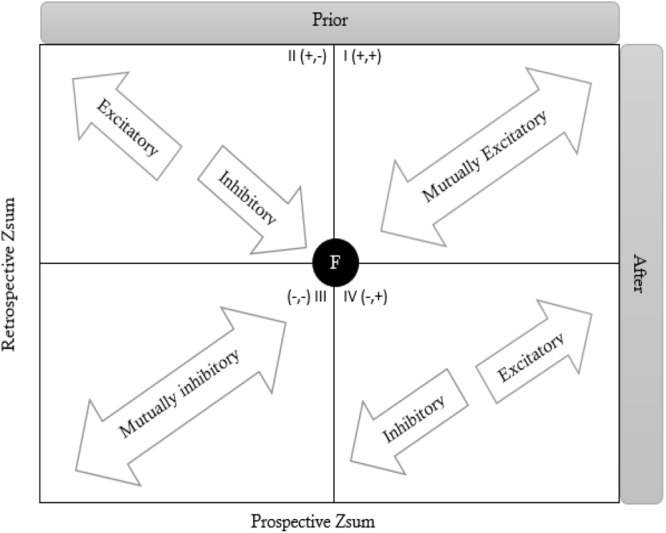
Graphic depiction of relationships between conditional and given behaviors in polar coordinate maps according to quadrant in which vector is located and their occurrence prior or after the focal behavior (F) (Modified from Castañer et al., 2016).

To extract information that can be used by coaches and psychologists, a specific analysis (e.g., polar coordinates analysis) needs to be carried out. The polar coordinate analysis aims to detect behavioral patterns between focal (behavior of interest) and conditioned behaviors (those that precede or follow focal behavior), which occur with a higher probability than by chance (Anguera, 2003, 2008). The analysis is carried out in two steps: the first matches frequency tables with conditioned (observed) and unconditioned probabilities (by chance) (González-Prado et al., 2015). When conditioned probabilities exceed unconditioned ones, excitatory relationships appear in a technique known as lag sequential analysis (Anguera, 2008). The second step involves creating a vector map of four quadrants (see Figure 1) containing information on both retrospective and prospective perspectives (Anguera, 2000, 2001). This technique has also been used as a data reductionism technique, synthesizing the existing relationships and isolating those that occur most frequently (Hernández-Mendo and Anguera, 1999; Gorospe and Anguera, 2000).

Polar coordinates have been used on the sports field in disciplines such as soccer, tennis, Basque pelota and taekwondo (Hernández-Mendo and Anguera, 1998; Castellano and Hernández-Mendo, 2003; Castellano et al., 2007; Perea et al., 2012; López-López et al., 2015; Menescardi et al., 2016). Specifically, only two studies have applied the technique to investigate the relationship between technical-tactical actions and the different ways male Olympic taekwondo athletes use to score (López-López et al., 2015; Menescardi et al., 2016). In particular, a relationship between anticipatory counterattacks (ACAs) and effective one- and two- point actions to the chest (López-López et al., 2015), and sequences of attacks followed by counterattacks actions (Menescardi et al., 2016) were revealed, in line with the peculiarity of this sport (Joseph, 2012).

However, these studies did not analyze the whole competition and did not consider differences that occur during its phases (Menescardi et al., 2014); in fact, the entire competition should be analyzed. The literature on Olympic taekwondo match analysis mainly focuses on the frequency of technical-tactical actions (González, 2011; Kazemi et al., 2014; Tornello et al., 2014; González-Prado et al., 2015), whereas scant information is given on the match sequences of elite male and female taekwondo athletes. In this regard, previous studies have shown that males performed more actions than females in the Olympics (Kazemi et al., 2009), elite (Kwok, 2012), college (Menescardi et al., 2012), or youth (Casolino et al., 2012) competitions. The differences between genders were pointed out, showing that females won more bouts by superiority than males. Also, more warnings were given to female medalists than non-medalists as compared to males, concluding that male athletes had a more dynamic fighting style than the females (Matsushigue et al., 2009). These results highlight the impossibility of extrapolating inter-gender data.

The aim of the study then was to analyze the relationships between technical-tactical behaviors (i.e., tactics, techniques, the kicking zone, the kicking leg, laterality, and guard) that occur before and after a point is scored (i.e., one, two, three, or four points) by introducing an observational methodology into the mixed methods framework. Based on the theoretical framework and literature, we expected to find technical-tactical patterns that would allow coaches and athletes to explain successful behaviors and also some of the differentiated patterns between female and male athletes. This analysis allows researchers to extract behavioral patterns suitable for use by coaches and psychologists to enable them teach effective tactics for competitions.

## Materials and Methods

### Experimental Approach to the Problem

The present study is based on an observational methodology inserted into the mixed method approach. Mixed methods involve the collection, analysis and combination of quantitative and qualitative data with rigor and have the flexibility required to study behaviors in a natural context (Anguera et al., 2014). The observational methodology collects data directly from training sessions and/or competitions and has the advantage of observing spontaneous behavior (Anguera and Hernández-Mendo, 2016). It non-intrusively assesses physical and psychological demands during competition (Anguera et al., 2014). The data can be used to build research models and for the purposes of physical and psychological training (Anguera et al., 2014). Specifically, polar coordinate analysis is a powerful technique for studying tactical behaviors in the field of sport (Aragon et al., 2017). Several studies have used this technique to analyze the relationships (Aragon et al., 2017; Maneiro and Amatria, 2018) between key behaviors and improve coaching decisions. It also enables coaches and psychologists to suggest both offensive and defensive strategies (Maneiro and Amatria, 2018). To test the hypothesis of technical-tactical patterns that explain successful behaviors, this study describes behaviors (i.e., tactics, techniques, kicking zone, kicking leg, laterality, and guard) that occur before and after points are scored (i.e., one, two, three, or four points) by a polar coordinates analysis. As applied to taekwondo, this provides information on the event before (QI and QII), and after scoring (QI and QIV) or on those that prevents (QIII) scoring.

### Methodology and Design

An observational methodology was used to collect and analyze data (Castellano and Hernández-Mendo, 2003; López-López et al., 2015). The N/S/M design was used in the present study (Arias-Pujol and Anguera, 2017), N refers to nomothetic (focusing on 128 athletes), S refers to inter-sessional follow-up (151 bouts were recorded) and intra-sessional follow-up (continuous recording of specific moves), and M refers to multidimensional (analysis of multiple criteria, or levels of response, using the purpose-designed TDKOT observational instrument).

### Participants and Sample

A total of 151 bouts (male; *n* = 75, and female; *n* = 76) in the Olympic tournament (2012 London Olympic Games) were coded (one male semi-final bout was not analyzed due to the injury of one athlete) from public television broadcasts. From these bouts a total of 24,940 actions (*M* = 173.3 actions per bout) were registered and codified. The action inclusion criterion was to have clear observability of each action (Castañer et al., 2016). When the action was unclear or impossible to fully identify, it was not codified. This research was carried out in accordance with the Declaration of Helsinki and the Belmont Report. A review by an ethics committee and written informed consents were not required in this study as: (a) it involved the observation of people in public places (competition area) where the athletes targeted for observation had no reasonable expectation of privacy; (b) it did not include any intervention by the researcher; and (c) it did not collect personal information disseminated through photographic, film or video footage in the research results (National Institutes of Health, 1978; American Psychological Association, 2002).

### Materials

To code the athlete’s behavior, the TKD observational tool, validated by Menescardi et al. (2017) was used (Table 1). This tool contains seven exhaustive category systems (criteria) and 24 mutually exclusive categories distributed within each criterion. HOISAN software (Hernández-Mendo et al., 2012) was used for codifying data and carrying out the polar coordinate analysis.

**Table 1:** Categories, codes and categorical core of the observational tool used.

Criteria	Categories	Code	Categorical core or description
Tactics	Block	BLO	Defensive actions to avoid the impact of a kick by placing one arm or leg between the protector and the leg of the opponent. This does not have a scoring objective.
	Dodge	DOD	Defensive actions to avoid the impact of a kick by placing one arm or leg between the protector and the leg of the opponent. This does not have a scoring objective.
	Cut	CUT	Defensive forward movement to avoid being beaten by a close opponent, and to prevent the attacking action from being completed. This does not have a scoring objective.
	Opening	APE	Movement to control the distance from the opponent or bridge the gap between both competitors.
	Direct attack	DIA	Offensive action with the objective of scoring, ending with impact on the opponent but without previous movement.
	Indirect attack	INA	Offensive action in order to score, ending with impact on the opponent and with previous movement such as a step, skip, opening, guard change, kicking trajectory modification, etc.
	Anticipated counterattack	ACA	Action that starts during the opponent’s attack with the purpose of scoring. The athlete kicks the attacker during the preparatory phase (guard) and/or initial phase (when the opponent’s knee is being raised).
	Simultaneous counterattack	SCA	Action that starts at the same time as the opponent’s attack and has a scoring purpose. The athlete kicks at the same time as the opponent. Thus, the counter attacker kicks at the end of the attacker’s initial phase (leg raised) or during the impact momentum (impact phase) of the attacker’s kick.
	Posterior counterattack	PCA	Action that begins after the opponent’s attack (during the descending phase, or when attacker’s leg touches the ground) with a scoring purpose. Athletes kick at the same time. This action (sometimes) includes a previous backward displacement to dodge the opponent’s attack.
Techniques	Linear	LIN	The kicking leg is directed toward the front of the opponent’s body with a pushing motion in an attempt to kick the opponent with the sole of the foot.
	Circular	CIR	The kicking leg is directed toward the opponent’s side, with a circular movement in an attempt to kick the opponent with the instep.
	Spin	GIR	Action performed with a previous rotation, at least 180° from the initial position, before kicking the opponent.
Height target	Trunk	CHE	Kick to permitted areas of the trunk.
	Head	HEA	Kick to permitted areas of the head.
Laterality	Right	RIG	Kick performed with the right leg.
	Left	LEF	Kick performed with the left leg.
Kicking leg	Front	FRO	Kick performed with the leg closest to the opponent.
	Back	BAC	Kick performed with leg furthest from the opponent.
Guard	Open	OPE	The front leg of each opponent differs (i.e., one of them has the left leg advanced and the other the right leg).
	Close	CLO	The front leg of both opponents is the same (e.g., both opponents have the left leg advanced).
Score	0 points	SC0	Action does not impact on the permitted areas, or impacts in these areas but not with enough force to score.
	1 point	SC1	Score obtained by a valid action performed to the trunk with a linear or circular technique.
	2 points	SC2	Score obtained by a valid action performed to the trunk using a spin beforehand.
	3 points	SC3	Score obtained by a valid action performed to the head with a linear or circular technique.
	4 points	SC4	Score obtained by a valid action performed to the head using a spin beforehand.

### Procedure

Each action (kick) was analyzed from the time the athlete’s foot started the movement, (i.e., raising the foot off the ground) until the kicking leg returned to the floor. Actions were analyzed by different, qualified observers; a procedure was developed for training observers (Menescardi et al., 2017) in accordance with the approach set forth in Anguera (2003). Six observers, divided into two groups (groups A and B), were involved in the reliability analysis of the data and evaluating inter-observer reliability. Each observer analyzed six combats. To evaluate intra-observer reliability, two observers analyzed the same six combats twice in a row. Cohen’s Kappa (κ) was used to calculate intra and inter-observer reliability. The inter and intra-observer results showed Cohen’s kappa values to be above 0.85, confirming an almost perfect conformity (Landis and Koch, 1977; Blanco-Villaseñor et al., 2014; López-López et al., 2015; Miarka et al., 2015). HOISAN v1.3.6.3 (Hernández-Mendo et al., 2014), was used to perform a lag sequential analysis of behaviors, followed by a polar coordinate analysis. HOISAN software integrates the analysis of the prospective (Sackett, 1980) and retrospective lag sequential analysis, specifically, the genuine retrospectivity (Anguera, 1997) of successive behaviors. The retrospective perspective of lag sequential analysis, in its genuine aspect, considers retrospectivity from the focal to the conditioned behavior. It considers negative lags and detects when conditional behaviors prior to focal behavior are revealed as preparatory to the occurrence of focal behavior (Anguera, 2008). The prospective perspective, however, is based on considering the conditioned behaviors as occurring subsequent to the focal behavior (Castañer et al., 2016). The relations between the behaviors were represented on a vector map on MATLAB software (Perea et al., 2012).

### Statistical Analysis

**FIGURE 2:**
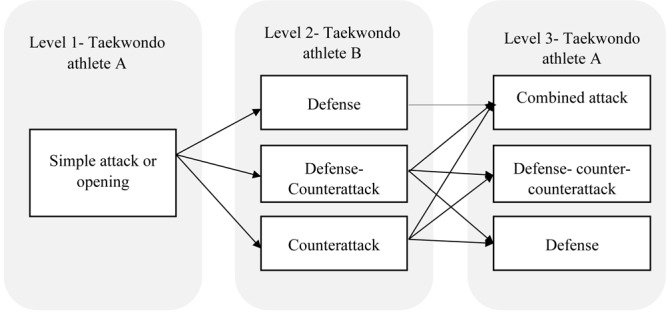
Offensive-defensive tactical schema in taekwondo (Menescardi, 2017).

Several negative (-1 and -2) and positive lags (+1 and +2) were used to identify behavioral associations between focal and conditioned behaviors. ±2 lags (corresponding to actions) have been used in previous works to determine the last and second-to-last actions prior to focal behavior (score) and the first two actions following the focal behavior (Anguera, 2008). Although, team sports studies normally use ±5 lags (Anguera et al., 2018), in adversary sports five actions before and after the focal behavior would not give a clear scenario of what occurs during a kicking sequence in competition. In combat sports it is more appropriate to use a restricted lag interval, as for example ±2 lags for the actions included in the tactical schema of taekwondo (Figure 2) as proposed by Menescardi (2017). The adjusted residual values obtained in the lag sequential analysis (excitatory and inhibitory actions) were then subjected to polar coordinate analysis (Sackett, 1980), considering both the retrospective and prospective perspectives.

The resulting polar coordinate maps show the associations between each focal behavior (as given behaviors are known in polar coordinate analysis) and all the conditional behaviors were analyzed (represented as vectors through the *Z*_sum_ parameter) (Santoyo and Anguera, 1993; Gorospe and Anguera, 2000). Every *Z*_sum_ parameter was understood as follows (Cochran, 1954; Sackett, 1980): $\mathrm{Zsum}\;=\frac{\Sigma z}{\sqrt{n}}$, where *n* represents the number of lags analyzed. Three main points should be considered when interpreting the associations: (a) the quadrant location of the vector; (b) the length or module of the vector; and (c) the angle (φ) of the vector with respect to the horizontal axis (Castañer et al., 2016, 2017; Arias-Pujol and Anguera, 2017). To calculate the length and angle of the corresponding vector and corresponding representation, the retrospective (*Y*-axis) and prospective (*X*-axis) *Z*_sum_ values for each conditional behavior are required (López-López et al., 2015; Arias-Pujol and Anguera, 2017). This determines the association between focal and conditioned behaviors. The length of the vector is calculated by the $\sqrt{{\left(\mathrm{ZsumProspective}\right)}^{2}+{\left(\mathrm{ZsumRetrospective}\right)}^{2}}$ (López-López et al., 2015; Arias-Pujol and Anguera, 2017). Finally, the angle is determined by dividing the retrospective *Z*_sum_ arcsine by the radius ($\varphi \;=\frac{\;arcsine\;of\;Y}{radius}$), giving rise to four relationships (López-López et al., 2015) according to the quadrant, in which the vector is located:

(a) Quadrant I (0-90°). Indicates that the focal and conditional behaviors are mutually activated in both perspectives; that is, the conditioned behaviors occur before and after the focal behavior (+, +).
(b) Quadrant II (90–180°). Indicates that the focal behavior inhibits the conditional behaviors but is also activated by them, that is, the conditioned behavior precedes but does not follow the focal behavior (+, -).
(c) Quadrant III (180-270°). Indicates that the focal and conditional behaviors are mutually inhibited, that is, the conditioned behavior neither precede nor follow the focal behavior (-, -).
(d) Quadrant IV (270-360°). Indicates that the focal behavior activates the conditional behaviors but is also inhibited by them, that is, the conditioned behavior does not precede but follows the focal behavior (-, +).

These relationships were represented with the focal behavior in the center of each vector map and the conditional behaviors in one of four quadrants. Although all the relationships appear on the vector representation of the polar coordinates map, only those whose module or radium vector length > 1.96 are considered significant (*p* < 0.05) (Hernández-Mendo and Anguera, 1999; Castellano and Hernández-Mendo, 2003; López-López et al., 2015; Arias-Pujol and Anguera, 2017; Castañer et al., 2017) and included in the results. A total of eight polar coordinates analyses were conducted, considering gender (two options: male or female) and the score criteria (four options: SC1, SC2, SC3, and SC4).

## Results

**FIGURE 3:**
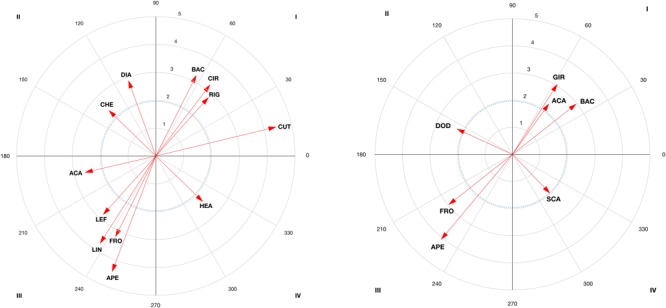
Representation of behavioral maps for SC1 category as focal behavior in female competitors **(Left)** and males **(Right)**. The behavioral map was represented divided into four quadrants, with each of the conditioned categories as vectors in the axis *X*-/*Y*- and their respective coordinates *Z*_sum_ prospective (*X-*) and *Z*_sum_ retrospective (*Y-*).

**FIGURE 4:**
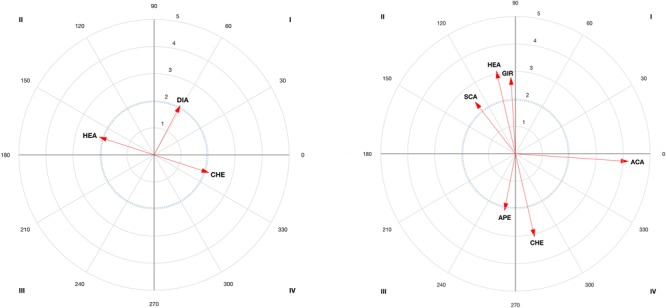
Representation of behavioral maps for SC2 category as focal behavior in female competitors **(Left)** and males **(Right)**. The behavioral map was represented divided into four quadrants, with each of the conditioned categories as vectors in the axis *X*-/*Y*- and their respective coordinates *Z*_sum_ prospective (*X*-) and *Z*_sum_ retrospective (*Y*-).

**FIGURE 5:**
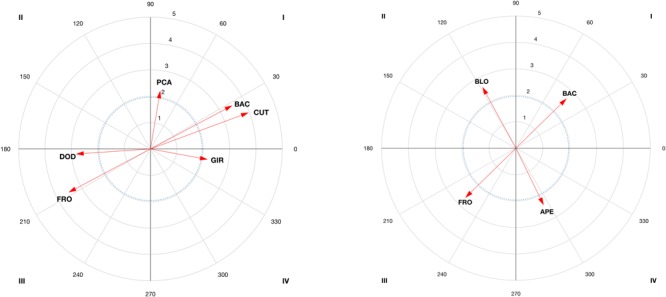
Representation of behavioral maps for SC3 category as focal behavior in female competitors **(Left)** and males **(Right)**. The behavioral map was represented divided into four quadrants, with each of the conditioned categories as vectors in the axis *X-*/*Y*- and their respective coordinates *Z*_sum_ prospective (*X*-) and *Z*_sum_ retrospective (*Y*-).

**FIGURE 6:**
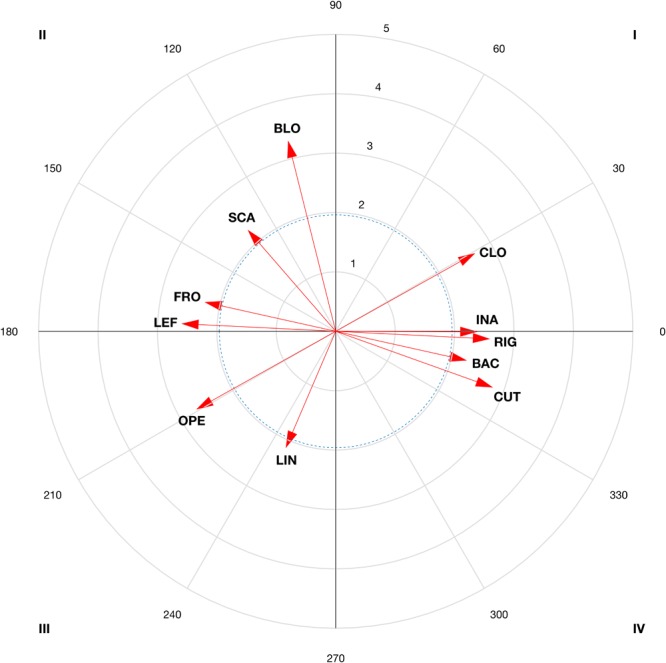
Representation of behavioral maps for SC4 category as focal behavior in male competitors. The behavioral map was represented divided into four quadrants, with each of the conditioned categories as vectors in the axis *X*-/*Y*- and their respective coordinates *Z*_sum_ prospective (*X*-) and *Z*_sum_ retrospective (*Y*-).

The results of the vector maps are shown in Figure 3–6 and the polar coordinates analysis for each category of the score criteria (as focal behaviors, i.e., SC1, SC2, SC3, and SC4) are shown in Tables 2–5. Females and males showed a total of 21 and 28 excitatory relationships between behaviors, respectively. For males, relationships were found in every focal behavior (SC1–SC4), however, for females, they were found in SC1, SC2, and SC3 (no relationship was found in SC4).

**Table 2:** Results of the polar coordinates’ analysis for SC1 focal category.

	Females						Males					
Cond.	Q.	Pros.	Retr.	Ratio	*R*	φ	Q.	Pros.	Retr.	Ratio	*R*	φ
BLO	IV	0.40	–0.57	–0.81	0.69	305.47	II	–0.33	1.07	0.96	1.13	107.18
DOD	II	–1.20	1.39	0.76	1.83	130.77	II	–2.02	0.93	0.42	2.22ˆ*	155.15
CUT	I	4.31	1.04	0.23	4.44ˆ*	13.55	IV	1.93	–0.33	–0.17	1.95	350.44
APE	III	–1.57	–4.14	–0.94	4.43ˆ*	249.25	III	–2.59	–3.13	–0.77	4.06ˆ*	230.44
DIA	II	–0.98	2.7	0.94	2.87ˆ*	110.00	II	–0.23	0.49	0.91	0.54	114.57
INA	II	–0.01	1.33	1.00	1.33	90.61	I	0.88	1.32	0.83	1.59	56.45
ACA	III	–2.54	–0.59	–0.23	2.61ˆ*	193.02	I	1.32	1.86	0.81	2.28ˆ*	54.59
SCA	IV	1.50	–1.13	–0.60	1.88	322.96	IV	1.36	–1.43	–0.72	1.97ˆ*	313.55
PCA	IV	0.23	–1.05	–0.98	1.07	282.20	I	1.17	0.39	0.31	1.24	18.33
LIN	III	–2.00	–3.13	–0.84	3.72ˆ*	237.43	III	–1.94	–0.12	–0.06	1.94	183.55
CIR	I	1.94	2.55	0.80	3.21ˆ*	52.70	IV	0.88	–1.36	–0.84	1.62	302.72
GIR	II	–0.07	0.86	1.00	0.86	94.72	I	1.63	2.58	0.85	3.05ˆ*	57.78
CHE	II	–1.68	1.63	0.70	2.35ˆ*	135.86	IV	0.60	–1.04	–0.87	1.20	300.04
HEA	IV	1.68	–1.63	–0.70	2.35ˆ*	315.86	II	–0.60	1.04	0.87	1.20	120.04
RIG	I	1.89	2.10	0.74	2.82ˆ*	48.04	III	–1.44	–0.91	–0.54	1.70	212.43
LEF	III	–1.89	–2.10	–0.74	2.82ˆ*	228.04	I	1.44	0.91	0.54	1.70	32.43
FRO	III	–1.45	–2.89	–0.89	3.24ˆ*	243.38	III	–2.31	–1.85	–0.63	2.96ˆ*	218.7
BAC	I	1.45	2.89	0.89	3.24ˆ*	63.38	I	2.31	1.85	0.63	2.96ˆ*	38.7
OPE	III	–0.86	–1.28	–0.83	1.54	236.24	I	0.84	0.85	0.71	1.20	45.24
CLO	I	0.86	1.28	0.83	1.54	56.24	III	–0.84	–0.85	–0.71	1.20	225.24

*^∗^Means significate vectors (radium > 1.96). Z_*sum*_ values, prospective (pros.) and retrospective (retr.), quadrants (Q), radium length (R), and ratio and vector angle (φ) are represented. BLO, blocks; DOD, dodges; CUT, cuts; APE, opening; DIA, direct attack; INA, indirect attack; ACA, anticipatory counterattack; SCA, simultaneous counterattack; PCA, posterior counterattack; LIN, linear technique; CIR, circular technique; GIR, spin technique; CHE, action to the chest protector; HEA, action to the head; RIG, right leg; LEF, left leg; FOR, forward leg; BAC, backward leg; OPE, open guard; CLO, close guard.*

**Table 3:** Results of the polar coordinates analysis for SC2 focal category.

	Females						Males					
Cond.	Q.	Pros.	Retr.	Ratio	*R*	φ	Q.	Pros.	Retr.	Ratio	*R*	φ
BLO	II	–1.52	1.05	0.57	1.85	145.46	IV	0.91	–0.4	–0.4	0.99	336.53
DOD	II	–0.80	0.13	0.17	0.81	170.46	III	–0.41	–1.2	–0.95	1.26	251.06
CUT	IV	0.42	–0.17	–0.37	0.46	338.2	IV	0.30	–0.3	–0.71	0.42	315.00
APE	IV	0.37	–0.81	–0.91	0.90	294.74	III	–0.40	–2.06	–0.98	2.10ˆ*	258.95
DIA	I	0.97	1.82	0.88	2.07ˆ*	62.03	II	–0.65	0.29	0.41	0.71	155.98
INA	III	–0.06	–0.96	–1.00	0.96	266.21	I	0.31	1.00	0.95	1.04	72.67
ACA	IV	1.25	–0.95	–0.6	1.57	322.87	IV	4.19	–0.28	–0.07	4.20ˆ*	356.23
SCA	III	–0.13	–0.83	–0.99	0.85	260.85	II	–1.50	1.90	0.78	2.42ˆ*	128.35
PCA	III	–0.04	–0.06	–0.87	0.07	240.95	I	0.24	0.70	0.95	0.74	71.05
LIN	III	–1.07	–0.93	–0.66	1.42	220.97	IV	0.13	–1.05	–0.99	1.06	276.89
CIR	I	1.04	1.37	0.80	1.72	52.85	III	–0.01	–0.62	–1.00	0.62	268.68
GIR	III	–0.02	–0.94	–1.00	0.94	268.71	II	–0.17	2.79	1.00	2.79ˆ*	93.49
CHE	IV	2.03	–0.66	–0.31	2.13ˆ*	342.05	IV	0.71	–3.02	–0.97	3.10ˆ*	283.18
HEA	II	–2.03	0.66	0.31	2.13ˆ*	162.05	II	–0.71	3.02	0.97	3.10ˆ*	103.18
RIG	IV	0.68	–0.73	–0.73	1.00	312.99	I	0.71	0.16	0.21	0.72	12.41
LEF	II	–0.68	0.73	0.73	1.00	132.99	III	–0.71	–0.16	–0.21	0.72	192.41
FRO	III	–1.70	–0.04	–0.02	1.70	181.19	III	–0.04	–1.29	–1.00	1.29	268.43
BAC	I	1.70	0.04	0.02	1.70	1.19	I	0.04	1.29	1.00	1.29	88.43
OPE	IV	0.86	–1.60	–0.88	1.82	298.36	II	–0.83	0.24	0.28	0.86	163.8
CLO	II	–0.86	1.60	0.88	1.82	118.36	IV	0.83	–0.24	–0.28	0.86	343.8

*^∗^Means significate vectors (radium > 1.96). Z_*sum*_ values, prospective (pros.) and retrospective (retr.), quadrants (Q), radium length (R), and ratio and vector angle (φ) are represented. BLO, blocks; DOD, dodges; CUT, cuts; APE, opening; DIA, direct attack; INA, indirect attack; ACA, anticipatory counterattack; SCA, simultaneous counterattack; PCA, posterior counterattack; LIN, linear technique; CIR, circular technique; GIR, spin technique; CHE, action to the chest protector; HEA, action to the head; RIG, right leg; LEF, left leg; FOR, forward leg; BAC, backward leg; OPE, open guard; CLO, close guard.*

**Table 4:** Results of the polar coordinates analysis for SC3 focal category.

	Females						Males					
Cond.	Q.	Pros.	Retr.	Ratio	*R*	φ	Q.	Pros.	Retr.	Ratio	*R*	φ
BLO	III	–0.33	–0.75	–0.91	0.82	246.09	II	–1.24	2.3	0.88	2.61ˆ*	118.30
DOD	III	–2.81	–0.19	–0.07	2.81ˆ*	183.89	IV	1.63	–0.84	–0.46	1.84	332.74
CUT	I	3.71	1.37	0.35	3.95ˆ*	20.32	II	–0.73	0.16	0.21	0.74	167.94
APE	IV	0.52	–1.44	–0.94	1.53	289.69	IV	1.03	–2.14	–0.90	2.38ˆ*	295.57
DIA	II	–1.66	0.15	0.09	1.67	174.89	II	–0.95	1.29	0.81	1.60	126.36
INA	I	1.55	0.45	0.28	1.61	16.05	III	–0.47	–0.07	–0.15	0.48	188.49
ACA	III	–0.86	–0.91	–0.73	1.26	226.60	III	–0.57	–0.11	–0.18	0.58	190.49
SCA	IV	1.03	–0.33	–0.31	1.08	342.04	II	–0.82	0.36	0.40	0.90	156.27
PCA	I	0.37	2.19	0.99	2.22ˆ*	80.48	IV	1.51	–0.34	–0.22	1.54	347.30
LIN	III	–1.11	–1.35	–0.77	1.75	230.58	III	0.00	–1.10	–1.00	1.10	270.00
CIR	II	–0.04	1.49	1.00	1.49	91.63	II	–0.78	1.43	0.88	1.63	118.57
GIR	IV	2.15	–0.40	–0.18	2.19ˆ*	349.56	IV	1.35	–0.71	–0.46	1.52	332.37
CHE	I	0.44	0.66	0.83	0.79	56.31	III	–0.25	–0.79	–0.95	0.83	252.65
HEA	III	–0.44	–0.66	–0.83	0.79	236.31	I	0.25	0.79	0.95	0.83	72.65
RIG	I	0.93	0.40	0.39	1.01	23.15	III	–1.22	–1.24	–0.71	1.74	225.50
LEF	III	–0.93	–0.40	–0.39	1.01	203.15	I	1.22	1.24	0.71	1.74	45.50
FRO	III	–3.09	–1.64	–0.47	3.50ˆ*	207.96	III	–1.87	–1.87	–0.71	2.65ˆ*	224.89
BAC	I	3.09	1.64	0.47	3.50ˆ*	27.96	I	1.87	1.87	0.71	2.65ˆ*	44.89
OPE	I	1.22	0.35	0.28	1.27	16.12	II	–1.24	0.15	0.12	1.25	173.20
CLO	III	–1.22	–0.35	–0.28	1.27	196.12	IV	1.24	–0.15	–0.12	1.25	353.20

*^∗^Means significate vectors (radium > 1.96). Z_*sum*_ values, prospective (pros.) and retrospective (retr.), quadrants (Q), radium length (R), and ratio and vector angle (φ) are represented. BLO, blocks; DOD, dodges; CUT, Cuts; APE, opening; DIA, direct attack; INA, indirect attack; ACA, anticipatory counterattack; SCA, simultaneous counterattack; PCA, posterior counterattack; LIN, linear technique; CIR, circular technique; GIR, spin technique; CHE, action to the chest protector; HEA, action to the head; RIG, right leg; LEF, left leg; FOR, forward leg; BAC, backward leg; OPE, open guard; CLO, close guard*.

**Table 5:** Results of the polar coordinates analysis for SC4 focal category.

Males						
**Cond.**	**Q.**	**Pros.**	**Retr.**	**Ratio**	***R***	**φ**

BLO	II	–0.80	3.20	0.97	3.29ˆ*	104.04
DOD	III	–0.18	–1.61	–0.99	1.61	263.72
CUT	IV	2.63	–0.94	–0.34	2.79ˆ*	340.33
APE	III	–1.38	–1.38	–0.71	1.95	225.00
DIA	II	–1.07	0.33	0.29	1.12	163.16
INA	IV	2.37	–0.01	0.00	2.37ˆ*	359.83
ACA	III	–0.56	–0.56	–0.71	0.79	225.00
SCA	II	–1.47	1.70	0.76	2.25ˆ*	130.91
PCA	IV	0.88	–0.1	–0.11	0.89	353.61
LIN	III	–0.83	–1.95	–0.92	2.12ˆ*	246.85
CIR	I	1.26	1.03	0.63	1.62	39.17
GIR	II	–0.84	1.38	0.85	1.62	121.39
CHE	I	0.84	0.94	0.75	1.26	48.18
HEA	III	–0.84	–0.94	–0.75	1.26	228.18
RIG	IV	2.58	–0.13	–0.05	2.58ˆ*	357.18
LEF	II	–2.58	0.13	0.05	2.58ˆ*	177.18
FRO	II	–2.19	0.49	0.22	2.25ˆ*	167.45
BAC	IV	2.19	–0.49	–0.22	2.25ˆ*	347.45
OPE	III	–2.33	–1.31	–0.49	2.68ˆ*	209.28
CLO	I	2.33	1.31	0.49	2.68ˆ*	29.28

*^∗^Means significate vectors (radium > 1.96). Z_sum_ values, prospective (pros.) and retrospective (retr.), quadrants (Q), radium length (R), and ratio and vector angle (φ) are represented. BLO, blocks; DOD, dodges; CUT, cuts; APE, opening; DIA, direct attack; INA, indirect attack; ACA, anticipatory counterattack; SCA, simultaneous counterattack; PCA, posterior counterattack; LIN, linear technique; CIR, circular technique; GIR, spin technique; CHE, action to the chest protector; HEA, action to the head; RIG, right leg; LEF, left leg; FOR, forward leg; BAC, backward leg; OPE, open guard; CLO, close guard*.

## Discussion

The main findings of the present study are the relationships extracted for each score in each gender. The results showed a total (females and males) of 49 significant excitatory relationships (25 before -QI and QII- obtaining a point, and 24 after -QI and QIV- gaining a point), and 13 that are inhibited before and after (QIII) gaining a point. Of the 25 excitatory relationships, 11 explain female successful and 14 male successful patterns. Females used to score a point after a cut, used to shorten the distance with the opponent, and after a direct circular attack with the back right leg to the chest. A high probability of scoring two points was also found after direct attacks to the head, while scoring three points is probable after a cut, or a posterior counterattack with the back leg. For females, there is little probability of scoring one and three points by dodging, opening, linear actions or anticipatory kicking counterattacks with the front leg. Males often score one point after a dodge and a spin or an anticipatory back leg kicking counterattack. Two points are scored after a simultaneous counterattack (SCA) during a spinning kick to the head, while three points are scored after a block or a kick with the back leg. In addition, four points were scored after a block or a SCA with the front left leg in close guard position. Finally, no scoring points are obtained either before or after an opening with the front leg or with a linear kick in an open guard position. The relationships found can help coaches and psychologists to plan strategies for winning points (in QI and QII) and avoiding those that prevent points from being scored (QIII). The discussion will focus on the relationships in QI, QII and QIII, which were found to be related to scoring points. The QIV was found to be irrelevant for scoring, in agreement with previous studies that also analyzed the results of interest (Castañer et al., 2017).

### Elite Female Athletes’ Relationships

A total of 21 excitatory relationships between focal and conditioned behaviors have been found. From those excitatory relationships, 11 occurred before gaining a point (QI = 9; QII = 2), 12 occurred after (QI = 9; QIV = 3), while seven were inhibited before and after gaining a point (QIII). From a technical-tactical perspective, there is a high probability of scoring a point (SC1) after a cut (CUT), and after a circular (CIR) direct attack (DIA), with the right (RIG) back leg (BAC) to the chest (CHE) (QI and QII). This is in line with previous studies that found a preference for direct actions and circular techniques (Menescardi et al., 2012; Menescardi and Estevan, 2017), right back leg and actions to the chest in competition (Tornello et al., 2014). A high probability of scoring two points (SC2) was found after a direct attack (DIA) to the head (HEA). This might be due to an effective spinning action to the chest to counteract attacks to the head. In this sport, both spinning actions and actions to the head are given an additional score for their difficulty of execution (World Taekwondo Federation, 2012), then athletes used to perform one in order to counterattack the other one. Finally, a high probability of scoring three points (SC3) was found after a cut (CUT), or a posterior counterattack (PCA) with the back leg (BAC). The frequent use of actions with the back leg could be due to their involving a longer path than front leg actions (Menescardi, 2017), thus making it possible to kick to the head, required to gain three points. The relationship with cuts and posterior counterattacks is due to this tactical aspect being used by female competitors to shorten the distance and counterattack by cutting and kicking to the opponent’s head.

Regarding the behaviors in QIII, in females an inhibitory relationship existed between the focal and conditioned behaviors. The probability of scoring one and three points (SC1 and SC3) after a dodge (DOD) and an opening (APE) or a linear (LIN) anticipation counterattack (ACA), with the front, left leg (FRO/LEF) is limited. This is due to the opponent avoiding a point being scored, for instance, by moving away or dodging (Fargas, 1990) or by performing an ACA with the front left leg.

An interesting interpretation of behaviors is when the behavior’s vector angle (φ) is close to the limit between quadrants (for instance, when the vector is in QIII close to QII or QIV). This is the case for the counter-attack action (ACA) to score one (SC1, see Figure 3) and a dodge (DOD see Figure 5) to score three (SC3), both are close to QII (193,2 and 183,9 degrees, respectively). The counterattacking action occurs when athlete use an underdeveloped strategy to score. In the case where the angle of the behavior would be placed in QII, an association between the score and the preceding counter-attack exist. Polar coordinates analysis can thus be used by coaches to examine whether the athletes’ behavior activates or inhibits his/her effectiveness according to the type of score and technical-tactical aspects. Training can thus be oriented toward developing new strategies used to defeat the opponent and void his/her attacks. Not only the length of the vector (the longer the vector the stronger the relationship expressed in *z*) but also the angle should be considered in polar coordinates. This is because in cases where the vector is close to changing the quadrant (less than 45 degrees to change to QII and QIV), researchers and sports teams could follow these behaviors to detect possible changes in behavioral tendencies (Castellano and Hernández-Mendo, 2003).

No significant relationship was found between technical-tactical actions and those that precede four-point score (SC4). This could be because females use heterogeneous methods to obtain four points. As the analysis did not find a pattern, the probability of obtaining four points was attributed to chance. A second explanation, as has also been suggested by previous studies (González, 2011; Menescardi, 2017), is that performing few actions allows them to obtain four points. Female athletes should thus create their own strategies and tactics according to their opponent’s strategy.

### Elite Male Athletes’ Relationships

In males, we found a total of 28 significant excitatory relationships, 14 before obtaining a point (QI = 5; QII = 9), 13 after scoring (QI = 5; QIV = 8) and six inhibited (QIII) before and after obtaining a point (21 relationships were found in females). This difference could be due to males using more behavioral patterns to score, while females use fewer patterns more frequently, indicating their different ways of competing and scoring, which could have implications for training (Menescardi et al., 2012).

From a technical-tactical perspective, males have a high probability of scoring one point (see Figure 3) after a dodge (DOD) and a spin (GIR) or an ACA with the back (BAC) leg. This is in line with previous studies (Kazemi et al., 2014) that highlighted that males use attacking actions to score one point (SC1), which contrasts with obtaining a point after a dodge, since the sequence is ended and a new sequence starts (with an attack). Other studies have pointed out the advantage of using the longer time needed to carry out a spin (Cheng et al., 2015; López-López et al., 2015) as compared to linear or circular kicks (Serina and Lieu, 1991). Actions with the back leg may possible unbalance the opponent (González, 2011) and have greater impact force (Pieter and Pieter, 1995; Pêdzich et al., 2006). This is important for achieving a point with the use of the electronic chest protector system. The possibility of scoring before and after an anticipated counterattack has also been pointed out in previous studies (Falco et al., 2014) due to its difficult timing. A high probability of scoring two points (SC2; see Figure 4) has been found after a SCA during a spinning kick (GIR) to the head (HEA). The scenario of performing spin-actions by both counterparts has been proven previously, showing spin-counterattacks as effective (López-López et al., 2015). The relationship with actions to the head could be explained by the necessity of increasing the score, not only by doing spin-actions but also performing actions to the head, as occurred in female bouts. A high probability of scoring three points (SC3; see Figure 5) was found after a block (BLO) or a kick with the back (BAC) leg. These results are especially relevant in clench situations (body–body situations) in which athletes try to continue the tactical sequence, chaining various actions with kicks to the head. The chaining of actions, although rarely used by male competitors (González, 2011), is revealed as an effective tactic if the opponent is distracted. Finally, a block is the behavior with the highest probability of scoring four points (SC4; see Figure 6), or a SCA with the front (FRO) left (LEF) leg in close guard position (CLO). The relationship found with blocks, actions performed with the front left leg and SCAs are revealed as favorable scenarios for spinning actions to the head and scoring four points. In addition, the relationship between scoring four points and close guard could be due to this being the most frequently used guard by males in competition (González, 2011; López-López et al., 2015).

Regarding the behaviors grouped in QIII, males obtained no scoring points either before or after an opening (APE) (SC1 or SC2, Figure 3, 4, respectively), with the front (FRO) leg (SC1 or SC3), or with a linear kick (LIN) in an open guard (OPE) position (SC4, Figure 6). This ‘no relationship with openings’, we speculate, reflects the use of those actions to test the opponent by performing movements like the *miro chagui*. This is usually performed with the front leg, which, by definition does not allow scoring (Menescardi et al., 2017) and therefore the relationship is in the third quadrant. The inclusion of linear actions in this quadrant (QIII) could occur because the circular and spinning actions already appear in QIV. Considering that only four points can be achieved by spin-action, in terms of probability, linear actions are infrequent before and after obtaining four points (SC4).

The present study has two limitations: the first is the fact that the results should be interpreted according to the characteristics of the polar coordinates analysis, in terms of the probability of each type of behavior, that is, the probability of one of the categories in the same criterion. This means, that if one category (i.e., the left leg) is found in one quadrant, the other (i.e., the right leg) should appear in another. This is because it is improbable that similar actions be performed before and after scoring (according to the exhaustive and mutually exclusive characteristics of the observational tool). It should also be noted that this type of analysis provides information on what happens before and after scoring (SC1, SC2, SC3, or SC4), but not on how the point was achieved. The lack of classifying data in relation to match outcomes (Gómez et al., 2016a,b) could also be seen as a limitation. Future studies should consider using the same methodology to analyze taekwondo performance including the discrimination of winning and losing close and unbalanced matches, which would provide more specific data and have valuable practical applications. It would also be interesting to analyze the different championship levels (world, national, or college) and the same level over time to monitor the influence of new rules and regulations and changes in refereeing practice and performance.

From a practical point of view, we suggest designing tactical strategies to train actions that permit scoring when detecting the actions that occurred before (QI and QII), as well as defensive (QIII). In addition, these results could be relevant for estimating and describing combat tactical patterns and their psychological effect (Hernández-Mendo and Anguera, 2001). In this line, sports psychologists agree on the importance of attitude, confidence, motivation and stress control through protocols of visualization of the technical-tactical aspects to enhance strengths and exploit the opponent’s weak points (Hernández-Mendo and Morales-Sánchez, 2010). For example, mental training is suggested to reduce uncertainty, tension and negative mental influences (pressure, anxiety, etc.). Confidence could also be enhanced by, for example, simulating training methods (Xiong, 2012). The results can also help in planning tactical training by mechanizing and automating tactical situations, as well as coping strategies for stress through or self-regulation work to make good decisions as well as relaxation work, imagery, attention and concentration training, self-instruction or positive reinforcement can be used to produce adaptive behaviors for motor improvement (Hernández-Mendo and Morales-Sánchez, 2010). In short, it is necessary to introduce competition situation models into training, for instance, taking into account the different scoring patterns used by males and females and to create specific strategies and tactics according to the opponent’s characteristics. Sport psychologists play an essential role in helping athletes reach their maximum sports performance (Orlick, 1996; Alvarez et al., 2014).

## Conclusion

Taekwondo research could also use the observational methodology described here which highlights the most important factors that affect the results of bouts. Sports researchers in other fields could also use this methodology and include the statistical techniques mentioned (i.e., the polar coordinate analysis) to provide objective data on competitive behavior. Future studies should investigate the relationships between behaviors according to the new regulations. Studies focusing on technical and tactical aspects of taekwondo could provide interesting and complementary insights into training aspects of this sport. For psychologists and coaches, it is important to discover the relationship between both technical and tactical actions in order to increase knowledge on interdependent behaviors and how they are produced. Psychologists and coaches can use the information obtained regarding the patterns that athletes use in competition to plan visualization sessions and tactical strategies. The results of the current study can be summarized as follows:

Effective actions to the trunk (SC1) were preceded and followed by spin actions with the back leg and anticipated counterattacks in males, while females opted to cut and performed circular techniques with the right back leg (QI). Direct attacks to the chest occurred before scoring in females and dodges in males (QII), while actions to the head in females and SCAs in males occurred after scoring (QIV). While there is probability of scoring one point in actions with the front leg and openings in both genders, anticipating counterattacks with a linear technique and the left leg in females is limited (QIII).

Effective spin-actions to the trunk (SC2) were preceded and followed by direct attacks in females (QI) and only preceded by actions to the head in both genders and SCAs in males (QII). Those behaviors were followed by actions to the trunk in both genders and anticipated in males (QIV). The probably of scoring two points when openings are performed is limited in males (QIII).

Effective actions to the head (SC3) were related to cut and posterior counterattacks with the backward leg in both perspectives and genders (QI) and cuts in retrospective in males (QII) and spin-actions in females and openings in males in prospective (QIV). The probability of scoring three points with front kicks in both genders and dodges, in females, is limited (QIII).

Effective spin-actions to the head (SC4) were carried out in a scenario of close guard (QI), preceded by blocks, actions with front right leg and SCAs (QII), followed by indirect actions with back and forward leg and cuts (QIV) in males. In SC4 and females, no relationships were found. The probability of scoring four points with open guard and linear kicks is limited in males (QIII).

Coaches may use these findings for specific tasks related to technical-tactical improvement of scoring effectiveness. Studies of this type could also be useful for establishing defensive strategies against specific actions. Sports psychologists could plan psychological intervention according to the athlete’s and his/her opponents’ characteristics. It would be interesting for future research to consider others types of contexts (world, national, and college championships) or match outcomes, to better discriminate between the motor ability patterns of successful and unsuccessful performances.

## Author Contributions

CM participated in the study design and data collection, conducted statistical analyses and contributed to the interpretation of the results, drafted the manuscript, and approved the final manuscript as submitted. CF, IE, and AH-M conceived the study, participated in its design and coordination, contributed to video coding, data collection and to the interpretation of results, drafted the manuscript, and approved the final manuscript as submitted. CR and VM-S participated in the study design, contributed to the interpretation of the results, reviewed and provided feedback to the manuscript, and approved the final manuscript as submitted. All authors made substantial contributions to the final manuscript.

## Conflict of Interest Statement

The authors declare that the research was conducted in the absence of any commercial or financial relationships that could be construed as a potential conflict of interest.
